# Facial Pilomatrixoma in a Child: A Rare Differential for a Soft Tissue Lesion

**DOI:** 10.7759/cureus.85989

**Published:** 2025-06-14

**Authors:** Muhammad Mudasir Saleem, Mishal Pervaiz, Uswah Shoaib, Ismail Mazhar, Mir Muhammad Rai, Saeed Ur Rehman, Kurrat Ul Aaien, Syed Atta Ur Rehman

**Affiliations:** 1 Pediatric and General Surgery, Combined Military Hospital, Lahore, PAK; 2 Anesthesiology, Punjab Rangers Teaching Hospital, Lahore, PAK; 3 Pediatric Surgery, CMH (Combined Military Hospital) Lahore Medical College and Institute of Dentistry, Lahore, PAK; 4 Pediatric Surgery, Loralai Medical College, Loralai, PAK; 5 Medical School, Bolan Medical College, Quetta, PAK

**Keywords:** benign tumor, hair follicle matrix, histopathological diagnosis, pilomatrixoma, surgical excision

## Abstract

Pilomatrixoma is an uncommon benign skin tumor originating from hair matrix cells, most frequently observed in children. Due to its slow growth and nonspecific presentation, it is often misdiagnosed as other skin or soft tissue lesions. A high index of suspicion is crucial for early recognition and appropriate management.

We report the case of an eight-year-old boy who presented with a gradually enlarging, firm, non-tender, mobile swelling on the right cheek over two years. The mass, located anterior and slightly inferior to the right ear near the angle of the mandible, had no associated history of trauma or systemic illness. Surgical excision under general anesthesia was performed. Histopathological examination confirmed the diagnosis of pilomatrixoma. No preoperative imaging was performed, as clinical assessment favored a benign superficial lesion. The patient recovered uneventfully, and no recurrence was observed during a six-month postoperative follow-up.

This case underscores the importance of including pilomatrixoma in the differential diagnosis of pediatric facial masses. While our follow-up duration was limited to six months, no signs of recurrence were noted during that period. Timely clinical recognition and complete surgical excision generally yield excellent outcomes, although longer-term surveillance is ideal.

## Introduction

Pilomatrixoma is a rare, benign tumor arising from hair follicle matrix cells, constituting only 0.001-1% of all skin lesions [1,2]. It most commonly affects children, especially female children, and typically presents as a solitary, firm, subcutaneous nodule on the head, neck, or upper limbs [3]. Despite being benign, its variable presentation often results in misdiagnosis as dermoid cysts, epidermal inclusion cysts, branchial cleft remnants, or even parotid tumors, particularly when located near the preauricular region.

The lesion in our case mimicked a parotid-tail mass because of its anatomical location, lack of skin discoloration or “tent sign,” and absence of trauma history. These features reduced suspicion of pilomatrixoma and could have led to more invasive surgical planning. Such diagnostic dilemmas underscore the importance of considering pilomatrixoma in the differential diagnosis of slow-growing pediatric facial masses. Although histopathology remains the gold standard, ultrasound can support diagnosis by detecting characteristic calcifications and excluding vascular or cystic lesions.

We report this case to highlight how atypical presentation may mislead clinicians, resulting in delayed or inappropriate management. The patient was successfully treated with surgical excision and remains recurrence-free on follow-up. This report aims to raise awareness about clinical features and diagnostic approaches that can improve early identification of this underrecognized tumor.

## Case presentation

**Figure 1:**
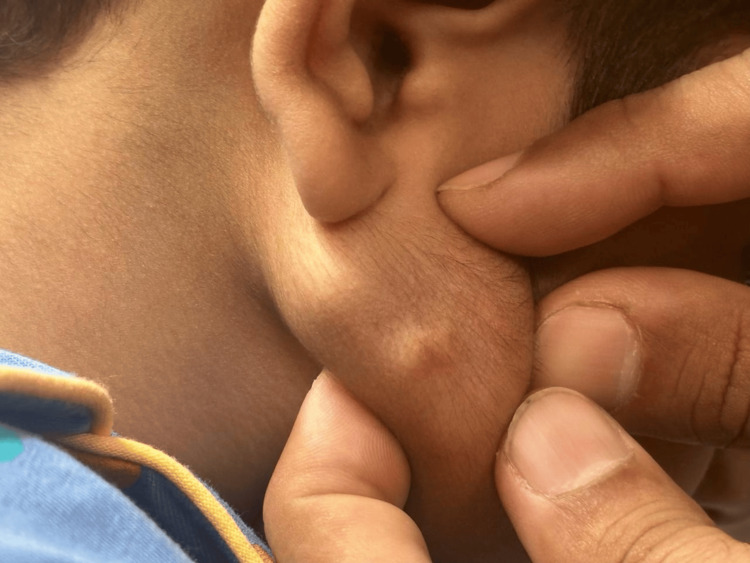
Preoperative presentation of the swelling on the right side of the face anterior to the mandible.

An eight-year-old boy attended our pediatric surgery clinic with swelling on the right pre-auricular region, ~1 cm anterior to the tragus and superficial to the tail of the parotid, overlying the mandibular ramus (Figure 1). His parents had first noticed the lesion two years earlier; it had grown steadily without pain or discharge. There was no history of trauma, vaccination, or systemic illness.

On inspection and palpation, the mass measured 2 cm × 2 cm, was firm, freely mobile in the subcutaneous plane, non-fluctuant, and non-tender. Mouth opening was normal (inter-incisal distance 40 mm); no trismus, dysphagia, or facial-nerve deficit was present. The overlying skin was intact, with no punctum or color change, and the “tent sign” was absent. The swelling did not enlarge with Valsalva, and cervical nodes were not palpable. The remainder of the head-and-neck and systemic examination was unremarkable. 

Baseline vital signs were within normal limits (temperature 36.8°C, heart rate 92 bpm, BP 104/64 mmHg, respiratory rate 18/min, SpO₂ 99% in room air). Laboratory tests (full blood count, electrolytes, renal profile, coagulation) were normal. High-resolution ultrasonography showed a well-defined, heterogeneously hyperechoic subcutaneous nodule with posterior acoustic shadowing suggestive of intralesional calcification; the parotid gland was uninvolved. Pre-anesthetic assessment classified the child as ASA I, and a single dose of intravenous cefazolin (30 mg kg⁻¹) was given 30 minutes before surgery.

**Figure 2:**
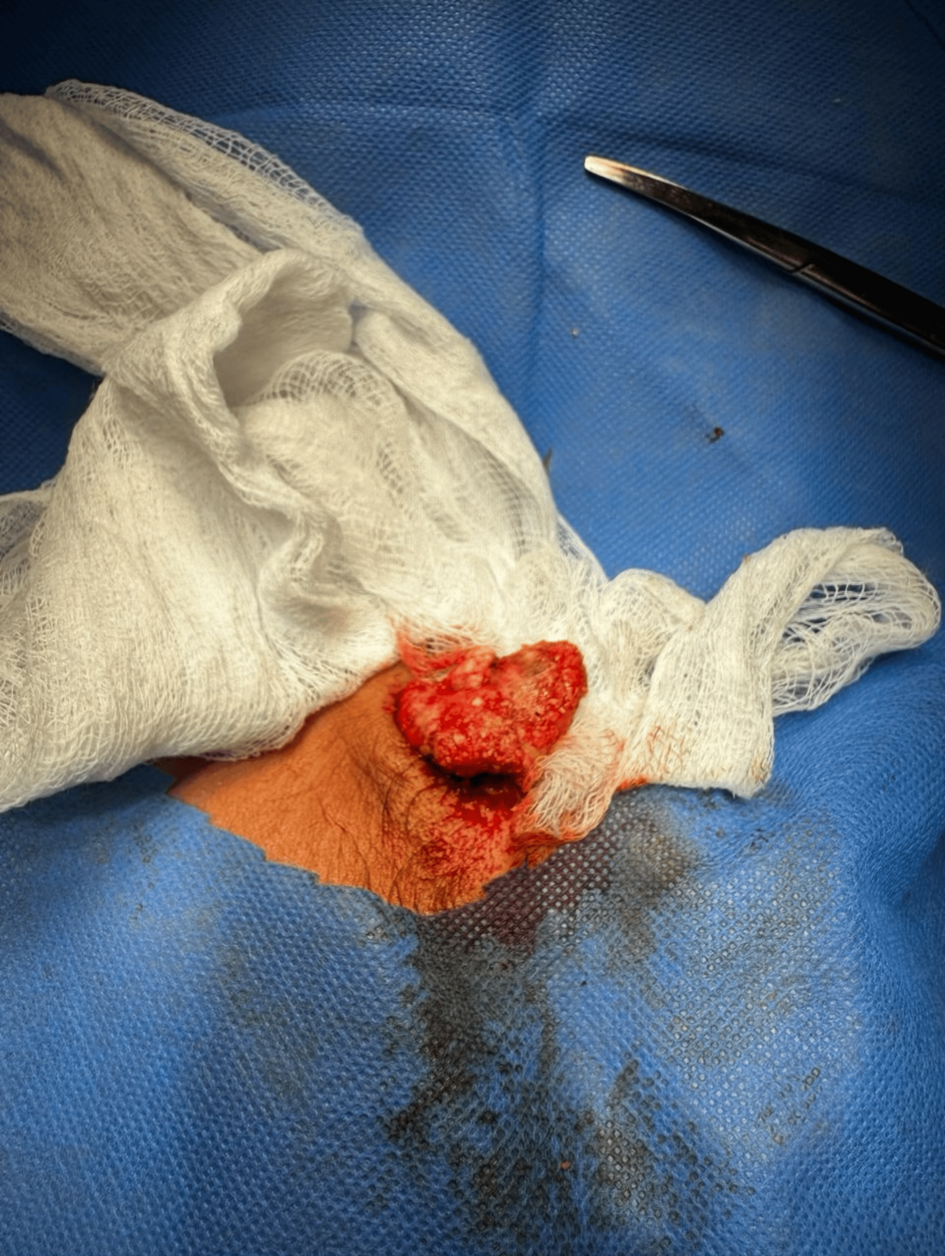
Perioperative appearance of the swelling having well circumcised borders, solid stroma, visible calcifications, and no gross vascular invasions.

**Figure 3:**
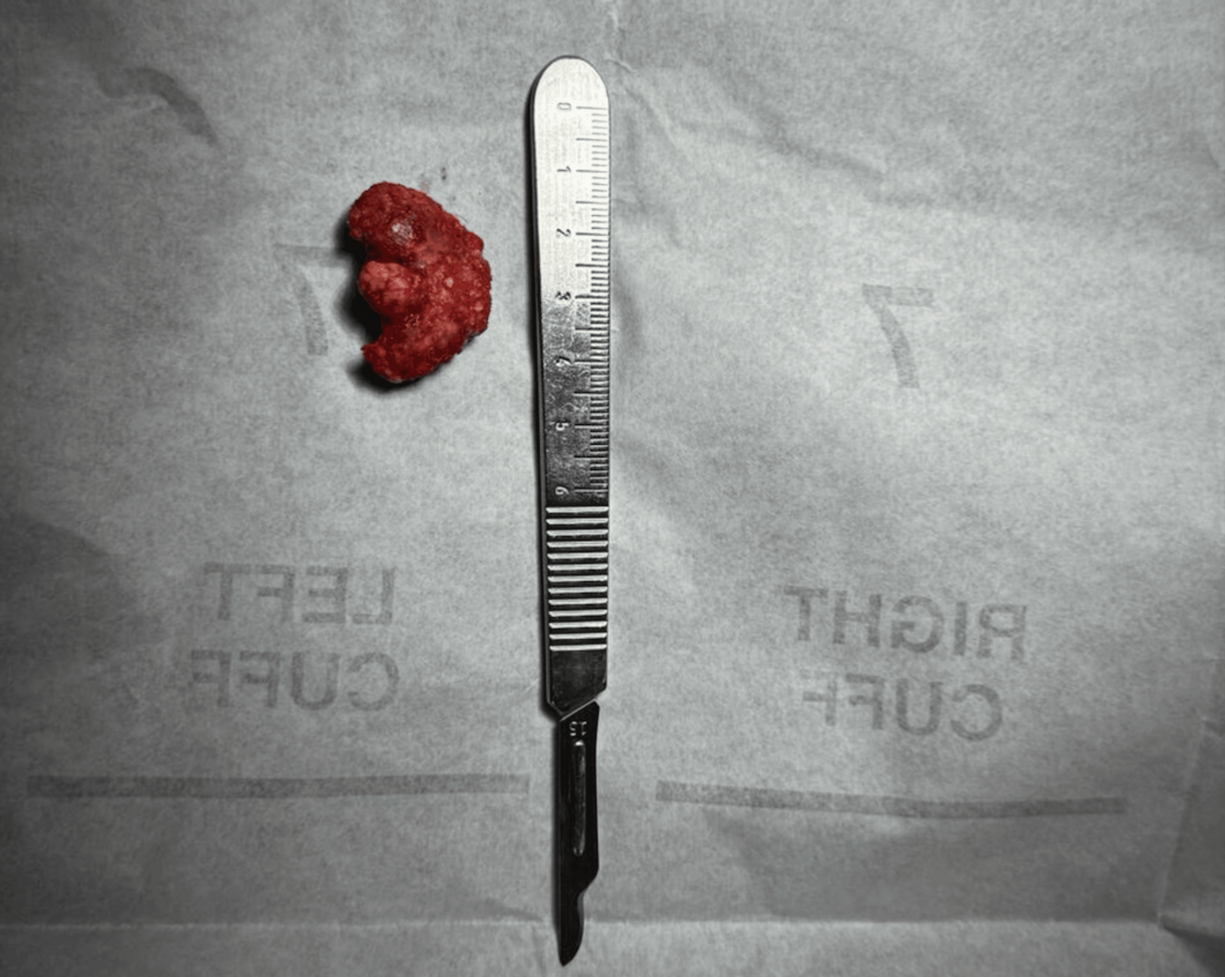
Completely excised mass measuring 3 cm x 2.5 cm.

**Figure 4:**
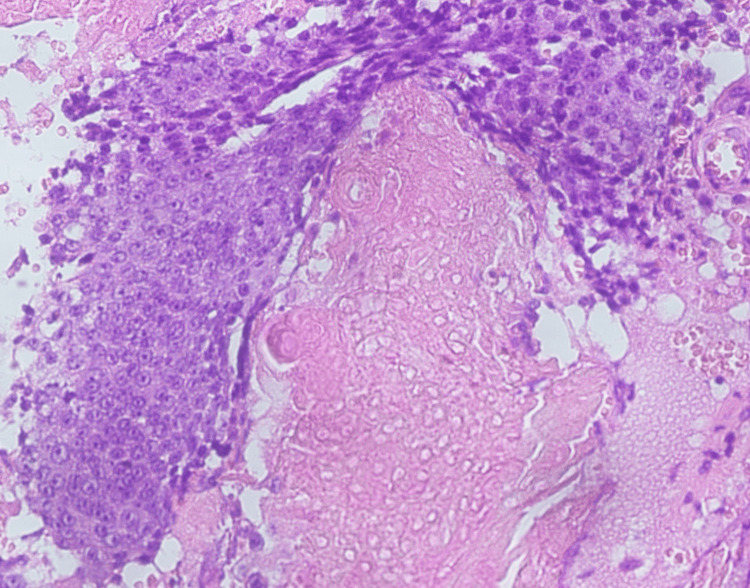
Hematoxylin and eosin (H&E) stain, original magnification ×400. The section shows basaloid cells with trichilemmal keratinization, ghost cell reaction, and calcification consistent with pilomatricoma.

Under general anesthesia, a transverse pre-auricular skin-crease incision was made. The lesion was dissected free and excised en-bloc with a narrow cuff of healthy tissue. Layered closure was performed with absorbable deep sutures and fine monofilament skin sutures. Histopathology revealed typical peripheral basaloid cells, transitional cells, and central anucleate “shadow” cells with focal calcification, confirming pilomatrixoma (Figures 2-4).

The postoperative course was uneventful and the patient was discharged on day 1 with simple analgesia. Follow-ups at one, three, and six months showed a flat, non-tender scar and no clinical or ultrasonographic evidence of recurrence. Early complete excision is therefore considered curative and prevents the (exceedingly rare) progression to malignant pilomatrixoma reported in the literature.

## Discussion

Pilomatrixoma typically presents as a solid, non-tender, subcutaneous nodule attached to the skin but not the underlying tissue. Tumors range from 0.4 cm to 20 cm, with a mean of 0.8 cm [4]. They grow slowly over months to years and exhibit a bimodal age distribution, with the highest incidence in the first two decades of life and a second peak between 50 and 65 years; among children, the most affected age group is eight to 13 years [5,6]. Our patient fits this epidemiological pattern yet demonstrates a diagnostically challenging feature; the mass lies immediately anterior to the tragus, an atypical site easily mistaken for a parotid‐tail lesion.

Common sites are the head, neck, and shoulders, though lesions may also occur on the chest, trunk, or lower extremities. These firm, mobile masses typically reside in the dermis and are usually asymptomatic, although they may be tender. The overlying skin is often normal yet can ulcerate or appear bluish or reddish, leading to diagnostic confusion with vascular lesions [7]. In addition, the classic “tent sign” was absent in our case [8], removing a helpful clinical clue and further increasing the likelihood of misdiagnosis.

Most pilomatrixomas are solitary; about 5% of children present with multiple lesions. The presence of six or more lesions has > 95 % specificity for an associated syndrome [9]. Because our child had a single, isolated lesion and no dysmorphic or systemic features, additional syndrome-oriented screening was unnecessary, illustrating the value of careful phenotype assessment before pursuing expansive genetic work-ups.

The pathogenesis of pilomatrixoma remains unclear, although trauma is implicated as a trigger. A review identified 21 cases arising at sites of prior trauma such as insect bites, dog scratches, injections, or vaccinations [10-13]. Our patient had no such antecedent, reinforcing the need for further research into alternative pathogenic mechanisms.

Pilomatrixoma is rarely included in the initial differential diagnosis. Common pre-operative considerations include epidermal inclusion cysts, dermoid cysts, branchial-cleft remnants, pre-auricular sinuses, foreign-body reactions, lipomas, and vascular tumors. In this context, point-of-care high-resolution ultrasonography proved decisive: the well-defined, heterogeneously hyperechoic nodule with posterior acoustic shadowing strongly suggested pilomatrixoma, excluded vascular malformation, and allowed us to plan a limited skin-crease excision rather than a more extensive parotid approach. Nevertheless, biopsy remains the gold standard for confirmation.

Histologically, pilomatrixomas exhibit a lobulated architecture with three distinct cell populations (peripheral basaloid cells, intermediate transitional cells, and central enucleated “shadow” cells), often accompanied by calcification [14]. All of these classic features were present in our specimen, confirming the diagnosis.

Malignant transformation is exceedingly rare: only 130 cases had been reported by 2017 [15]. Complete surgical excision with clear margins is curative, and the reported relapse rate is 0.3 %, usually due to incomplete removal [16]. Our patient underwent en-bloc excision with clear margins and remains disease-free six months post-operatively, underscoring that early ultrasound-guided recognition and cosmetically placed complete excision are sufficient for cure while avoiding unnecessary parotid surgery.

## Conclusions

Pilomatrixoma, though benign, can masquerade as a parotid-tail mass when it arises in the pre-auricular region. Point-of-care ultrasound in our patient demonstrated a superficial calcified nodule with no intraparotid extension, allowing a short skin-crease excision instead of a formal parotidectomy. Histology confirmed pilomatrixoma, and the child remains recurrence-free with an excellent cosmetic result at six months; annual clinical reviews are scheduled. This case underscores that early ultrasound-guided recognition and cosmetically placed complete excision are sufficient for cure and help avoid overtreatment in paediatric facial swellings.
